# Health Recommender Systems: Concepts, Requirements, Technical Basics and Challenges

**DOI:** 10.3390/ijerph110302580

**Published:** 2014-03-03

**Authors:** Martin Wiesner, Daniel Pfeifer

**Affiliations:** Department of Medical Informatics, Heilbronn University, Max-Planck-Str. 39, Heilbronn 74081, Germany; E-Mail: daniel.pfeifer@hs-heilbronn.de

**Keywords:** health recommender systems, information needs, personal health record, personalized medicine, health information seeking, Internet, relevance computation

## Abstract

During the last decades huge amounts of data have been collected in clinical databases representing patients' health states (e.g., as laboratory results, treatment plans, medical reports). Hence, digital information available for patient-oriented decision making has increased drastically but is often scattered across different sites. As as solution, *personal health record systems* (PHRS) are meant to centralize an individual's health data and to allow access for the owner as well as for authorized health professionals. Yet, expert-oriented language, complex interrelations of medical facts and information overload in general pose major obstacles for patients to understand their own record and to draw adequate conclusions. In this context, *recommender systems* may supply patients with additional laymen-friendly information helping to better comprehend their health status as represented by their record. However, such systems must be adapted to cope with the specific requirements in the health domain in order to deliver highly relevant information for patients. They are referred to as *health recommender systems* (HRS). In this article we give an introduction to *health recommender systems* and explain why they are a useful enhancement to PHR solutions. Basic concepts and scenarios are discussed and a first implementation is presented. In addition, we outline an evaluation approach for such a system, which is supported by medical experts. The construction of a test collection for case-related recommendations is described. Finally, challenges and open issues are discussed.

## 1. Introduction

Increasing health information needs and changes in information seeking behavior can be observed around the globe [1]. According to recent studies 81% of U.S. adults use the Internet and 59% say they have looked online for health information regarding diseases, diagnoses and different treatments [2]. Such effects influence the patient-physician relationship as educated patients raise questions or discuss treatment options [3,4,5]. Thus, patients tend to become active participants in the decision-making process. This change in the way of thinking is often referred to as patient empowerment [6,7].

However, information overload and irrelevant information are major obstacles for drawing conclusions on the personal health status and taking adequate actions [8]. Faced with a large amount of medical information on different channels (e.g., news sites, web forums, *etc.*) users often get lost or feel uncertain when investigating on their own. In addition, a manifold and heterogeneous medical vocabulary poses another barrier for laymen [9]. Therefore, improved personalized delivery of medical content can support users in finding relevant information [10,11,12].

Medical information available for patient-oriented decision making has increased drastically but is often scattered across different sites [13]. As as solution, *personal health record systems* (PHRS) are meant to centralize an individual's health data and to allow access for the owner as well as for authorized health professionals [14].

*Recommender systems* (RS) suggest items of interest to users of information systems or e-business systems and have evolved in recent decades. A typical and well known example is Amazon's suggest service for products. We believe the idea behind recommender systems can be adapted to cope with the special requirements of the health domain.

### 1.1. Outline of This Article

This paper contributes to the current state of research by discussing major concepts and challenges revolving around health recommender systems: In following section we give an informal definition of the term “health recommender system” followed by the presentation of an integrated system architecture, which relies on the existence of a PHRS. Moreover, we distinguish usage scenarios for two major user groups (*i.e.*, laymen and health professionals). In Section 2, we give an overview of the many areas that affect our contribution ranging from research in the field of health information seeking via foundations in computer science over to related systems of other researchers. Section 3 details on the requirements to be met for good recommendation quality—many of them incurring natural language processing of PHR entries, which often consist of semi-structured text. The section also introduces our prototypical implementation with its major components as well as its related processing steps. Section 4 describes our evaluation approach: As a basic step we must obtain a gold standard for optimal recommendations under a fixed setting. This will be done by conducting a specialized online survey amongst medical experts. We outline the design of a statistical test in order to compare results of our prototype to those of a naive implementation and to the expert's recommendations. Since the gold standard is not yet finished, Section 5 presents early results for typical recommendation cases. The examples indicate that our system may indeed return relevant recommendations for potential users. The paper closes with a conclusion and a reference to future work.

### 1.2. Definition & Typical Scenarios

*A health recommender systems* (HRS) is a specialization of an RS as defined by Ricci *et al.* ([15], p. 28). In the context of an HRS, a recommendable item of interest is a piece of non-confidential, scientifically proven or at least generally accepted medical information, which in itself is not linked to an individual's medical history. However, an HRS's suggestions are driven by individualized health data such as documented in a *personal health record* (PHR). According to [16] this source of information is considered the user profile of an *recommender system*.

The goal of an HRS is to supply it's user with medical information which is meant to be highly relevant to the medical development of the patient associated with that PHR. Related medical information may be recommended to health professionals who work on or with the given PHR but also it may be recommended to laymen inspecting their own PHR. Depending on a user's medical expertise an HRS should suggest medical information, which is comprehendable to that user.

**Figure 1:**
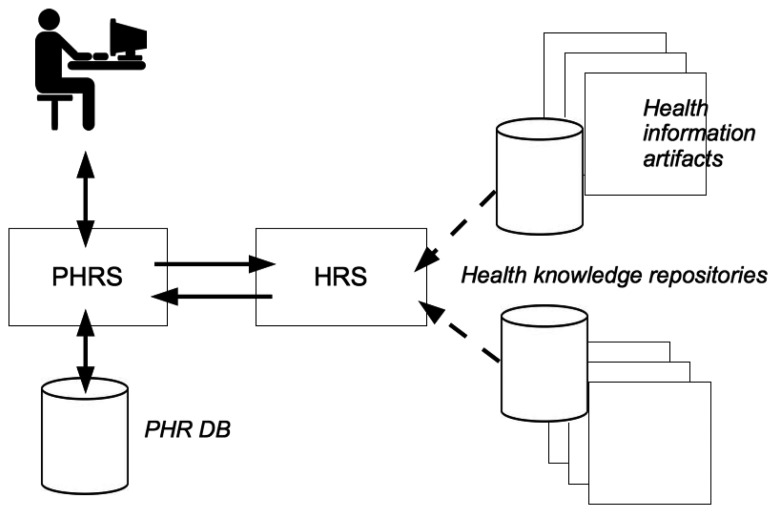
System context of an HRS-enabled PHR system.

For a successful integration into any health related information system, it is important to consider the system context of an HRS. As depicted in Figure 1, a profile-based HRS component is implemented as an extension of an existing PHR system. Data entries in a PHR database (DB) constitute the medical history of a PHR owner. Supplied with medical facts, an HRS computes a set of potentially relevant items of interest for a target user (e.g., a PHR owner or an authorized health professional). Such items originate from trustworthy health knowledge repositories and may be displayed while he/she inspects the PHR online.

Thus, it is possible to compute and deliver potentially relevant information items from trusted health related knowledge repositories. Depending on the expert level of a PHR user, at least two separate use cases can be defined as follows:

1. Use case A = Health professional as end-user
  In this scenario an HRS is used by a health professional to retrieve additional information for a certain case. For instance, related clinical guidelines or research articles from Pubmed (see: http://www.ncbi.nlm.nih.gov/pubmed) can be computed automatically (see Section 2.1). This form of case-related information enrichment might support a physician with the process of clinical diagnostics as latest research results can be used for decision support.
  In addition, laymen-friendly documents can also be retrieved for the purpose of a direct handout (*i.e.*, as a printout) to a patient when he or she is in a doctor's office for consultation. Thus, a user can be supplied with high quality information to cope with a certain disease or adapt his or her lifestyle habits.
2. Use case B = Patient as end-user
  In this scenario a layperson interacts with a HRS-enabled PHR without direct support by a physician. The system computes laymen-friendly content according to the person's longterm individual medical history. The relevant items are presented within the PHR system's user interface. By selecting the highest ranking documents or media content a patient is empowered in terms of health information acquisition. Thereby, the risk to retrieve “incomplete, misleading and inaccurate” content via famous search engines could be lowered (see Section 2.2), as we intend to recommend only evidence based (*i.e.*, high quality) health related content to end-users.
  Both scenarios intend to lower the effects of information overload which originate from the increasing amount of health related data [8,13]. For this article we focus on the patient-centered scenario. Hence, our first prototype of an HRS implementation will suggest laymen-friendly content only, *i.e.*, it realizes use case B.

## 2. Related Research

Recent trends in health information seeking and developments in the fields of personal health records [17,18] motivate our proposed approach for *health recommender systems*. Different methods exist to compute personalized medical recommendations. Many of them use methodologies and techniques originating from the fields of Information Retrieval and research on Recommender Systems. Hence, these fields are discussed in this section as well.

### 2.1. Electronic and Personal Health Records

Electronic Medical Records (EMRs) and Electronic Health Records (EHRs) provide the technology for the electronic storage of medical data and enables hospitals and other healthcare players to share such data electronically among authorized caregivers.

Personal health record (PHR) systems enable users to keep track of their own health data [14], most of which is still provided by health professionals. Hence, a longterm individual medical history can be carried together for an individual. The Markle Foundation [19] defines a PHR as follows:

A PHR is an electronic application through wich individuals can *access, manage* and *share* their health information, and that of others for whom they are authorized, in a private, secure and confidential environment.

Obviously, such a system is only useful to a patient if it provides more than just storing data such in structured record (name, age, diagnosis codes, laboratory results, *etc.*). The International Organization for Standardization (ISO) has defined four general PHR categories ([20], p. 14):

1. a self-contained EHR, maintained and controlled by the patient/consumer;
2. the same as 1. but maintained by a third party such as a web service provider;
3. a component of an ICEHR (Integrated Care EHR) maintained by a health provider (e.g., a general practitioner) and controlled at least partially by the patient/consumer;
4. the same as 3. but maintained and controlled completely by the patient/consumer

Patients can benefit if a PHR system supports them to view or act upon medical data and at the same time enables them to make informed decisions. It could also be a great value to patients if they are provided with PHR tools that enable them to communicate with caregivers in a direct way [21]. Thus, PHRS could “transform the tradition of episodic care to a more continuous communication channel between physicians and patients” [22].

Patients perceptions of the innovation characteristics of PHR systems are very important to PHR adoption. The innovative concept behind these systems implies that it is crucial for researchers to understand consumer perceptions on barriers to use such systems. Potential barriers to the adoption have been identified: data ownership, privacy, security, interoperability, PHR literacy and health literacy in general [14,23,24].

As health care professionals still play the primary role in the patient-physician relationship it is also important to consider their perspective when designing PHR systems. Huba and Zhang have found that PHR concepts are still a novel concept to many physicians [25]. Moreover, doctors suggested that PHR systems should support them in “knowledge discovery” (see Section 1.2, use case A), as well as to support in sharing patient information. In this context, Huang *et al.* used such a medical recommendation approach “to solve the information-overload problem by suggesting knowledge items of interest to clinicians” [26].

We believe that enabling knowledge discovery could be provided by a *health recommender system*. For this reason, it could a valuable add-on to existing PHR systems. Thus, both sides of the patient-physician relationship could benefit from individualized or case-related recommendations.

### 2.2. Health Information Seeking & Online Resources

Finding relevant information can be a challenging task, which is especially true for health related information. People's needs to search for individual health related information is often referred to as health information seeking [27,28]. In particular, such activities involve “any non-routine media use or interpersonal conversation about a specific health topic and thus includes behaviors such as viewing a special program about a health-related treatment, using a search engine to find information about a particular health topic on the Internet, and/or posing specific health-related questions to a friend, family member, or medical practitioner outside the normal flow of conversation” [29].

Powel *et al.* found at least four motivations why people use the Internet to find relevant content: “desires for reassurance, for second opinions for greater understanding of existing information and to circumvent perceived external barriers to traditional sources” [30]. Often, well known search engines or web-based encyclopedias provide a first starting point for laymen. Nevertheless, several barriers in terms of accessibility exist [31]:

- Findability: A user may have access to the Internet, yet it might be hard to determine which resource is relevant. Many times, a user is not even capable of finding suitable search terms to narrow the search space for his or her specific medical problem. Moreover, relationships between medical concepts are hard to understand, even if hyperlinks give a direction to another online resource. Even worse, the search result is not fully explored by most laymen, as “users of the Internet explore only the first few links on general search engines when seeking health information” [32].
- Comprehendability: Another problem arises from incomplete understanding of medical terms for people which lack medical knowledge. If a layperson has no mental model associated with some piece of information the value of it might be questionable. Particularly when a medical issue is involved, medical terminology used in many resources online is often misread or misinterpreted by laymen.
- Context Awareness: Health related information must be given or interpreted within a certain medical context, often in conjunction with a case or medical condition. Using a search engine or encyclopedia holds the risk of missing this kind of context. In turn, this may lead to erroneous conclusions and inadequate decisions.

As *health recommender systems* are meant to deliver context-related high quality content (see Section 1.2), they could be helpful in overcoming the aforementioned barriers. In particular, a semantically-enabled HRS could deal with complex relationships between medical concepts, resolve medical abbreviations and classification codes and adapt to the user's medical level of expertise. Such a system could also reduce the effects of information overload (*i.e.*, delayed decision making, distraction, waste of time and stress [33]), as it provides a user with those items of interest most relevant for a given case or the current medical context.

#### Health Information Artifacts

Medical knowledge available online has increased drastically during the last decades. Many articles and an increasing amount of digital media content (*i.e.*, video content) are available on health related websites. Such *health information artifacts* (HIA) are found in different information channels and can be obtained by various resources. Some resources mainly focus on health professionals (e.g., Pubmed), yet others are more consumer-centric (e.g., MedlinePlus (see: http://www.nlm.nih.gov/medlineplus), InformedHealthOnline (see: http://www.informedhealthonline.org) or WebMD (see: http://emedicine.medscape.com)) and the vocabulary in such resources differs from expert-centric ones. Consumer-centric medical content is often freely available and may consist of:

- expert-proven advisory on how to cope with a disease
- disease definitions in general which support in understanding medical terminology
- care plans which might prevent patients from acting against rules suggested by evidence-based medicine
- hints on healthier living or diet information

All these facets can be found in consumer-centric content. However, critics question the quality of such content. They consider such content as being “incomplete, misleading and inaccurate” ([34], p. 1244) or incomplete and not evidence-based [35]. Sonnenberg argues that “Most people will be unable to determine the qualifications of Web authors and separate truth from opinion” and “even well-educated users are unlikely to have the background required to critically evaluate medical information” ([36], p. 152). By contrast, according to the Health on the Net (HON) Foundation a large and growing majority of Internet users are concerned about quality of health information found on the Internet [37].

As a consequence, an HRS should only recommend health related content to end-users which was written by expert authors in a medical field. Moreover, it should be verified that a content provider has subscribed to the HONcode-principles, as it “demonstrates the intent of a website to publish transparent information” [38].

### 2.3. Methods from Computer Science (CS)

The detection of health information needs can be achieved by either analyzing PHR data entries or user originated search queries or by tracking of the browsing history of a user. Given a health context, many methods from the field of CS can be used to compute highly relevant recommendations. The following two sections briefly discuss related concepts.

#### 2.3.1. Information Retrieval (IR)

IR is a special research field of computer science that emerged in the 1970's. It addresses the human need to automatically find or filter relevant text documents from a potentially huge collection of managed documents. For this purpose the user is expected to describe its information interest via a query—usually a small list for search terms. A related IR system is meant to return many (and if possible all) but only those documents that comply with the user's corresponding information interest. This problem is also known as the *classical information retrieval problem* as it saves the user the work of manually searching the document collection.

Traditionally IR systems rely on word statistics regarding managed documents. In this context, the quality of a match between a user's query and a document is determined via the frequency of (certain) words in the document whereas the order of words in the document is (almost) ignored. This so-called *bag of words model* of IR incurs an enormous simplification. Nevertheless, experience shows that the bag of words model works quite well in practice when solving the classical IR problem.

Modern IR systems perform *document ranking*, which means that the quality of a match between a query *Q* and a managed document *D* is assessed via a *similarity function sc. sc* returns a real valued number ≥ 0 and, the larger *sc*(*Q,D*) the higher the degree of similarity. If *sc*(*Q, D*) = 0 then *Q* and *D* are considered completely dissimilar. At query-time, the IR system efficiently retrieves those documents *D_i_* from the collection, for which *sc*(*Q*, *D_i_*) > 0 hold while ignoring others. The retrieved documents are then ranked (*i.e.*, ordered) on the basis of decreasing *sc*-values. Typically, the *k* best-ranked documents are returned to the user (e.g., with *k* = 10).

There are numerous methods to compute *sc*(*Q,D*) [39,40]. One of the oldest and widest spread frameworks for this is Salton's *vector space model* (VSM) [41]. When using the VSM, *Q* and *D* are transformed (at least conceptually) into vectors *υ*(*Q*) and *υ*(*D*) of a real-valued *n*-dimensional vector space *V*, which holds as many dimensions as there exist unique terms when considering all managed documents. Thereby, a term is a delimited character sequence, which usually represents a potentially inflected word as extracted from at least one of the managed documents. Thus, every unique and extracted term of the document collection is represented by a dimension in *V* —in practice this can lead to thousands of dimensions. After the transformation of *Q* and *D*, a vector similarity measure is applied on *υ*(*Q*) and *υ*(*D*), e.g., by using the *scalar product* or the *cosine-similarity*. Regarding the transformation from *Q* to *υ*(*Q*) and *D* to *υ*(*D*), there also exist different ways to do this within the VSM. A more popular one is the so-called *tf-idf embedding* as first introduced in [41].

IR relies on *term matching* for document ranking, which means that *sc*(*Q, D*) generally increases with more terms appearing in both, *Q* and *D*. At the simplest level, a match between two terms from *Q* and *D* is based on exact match—meaning that their character sequence must be exactly identical. However in practice, this is usually too strict. e.g., if a user types “eye” in its query but the managed documents only contain the term “eyes”, then no results would be returned. There exist different approaches to overcome this problem:

- *Stemming and lemmatizing*: Assuming a term is an inflected word, that word is mapped to its root form. The mapping is performed on search terms as well as on document terms and the term matching is run afterwards. Referring to the example from above, “eyes” in related documents would be mapped to “eye” and so the query term “eye” would match for those documents. Stemming is usually rule based and a simplified but linguistically imprecise way to find the root of an inflected word [42,43]. Lemmatizing on the other hand, performs the mapping in linguistically precise way. As a drawback, lemmatizing usually requires a dictionary of a language comprising all (inflected) word forms associated with their root form. The dictionary helps to correctly map to a root form even in cases of irregular inflexion, which are frequent in many natural languages.
- *Query spell correction*: Typos in search terms can also lead to no matches or false matches on the document side. Spell correction procedures assess some sort of spelling distance between document terms and search terms. Reasonably close terms from the query side and the document side are then still considered as a term match. The so-called *editing distance* or *Levenshtein distance* is a common measure for this. Another basic one, which can be computed more efficiently, is based on the Jaccard index of the letter-based bi-gram or tri-gram sets of the terms under consideration [44]. e.g., the bi-gram set of the term “eye” is *A* = {“*ey*”, “*ye*”} and the bi-gram set for “eyes” is *B* = {“*ey*”, “*ye*”, “*es*”}. The Jaccard-index *J*(*A,B*) = |*A* ∩ *B*|/|*A* ∪ *B*| is a general measure for the similarity of two sets *A, B* and can vary between 0 (as completely dissimilar) and 1 (as identical). For the example from above, one obtains *J*(*A,B*) = |{“*ey*”, ”*ye*”}|/|{“*ey*”, “*ye*”, “*es*”}| = 2/3 which indicates a rather high degree of similarity.
- *Query expansion/query reformulation:* If a user searched for “iris” but instead, only the semantically related term “eye” occurred in managed documents, then the IR system would return no results. In this case, it may help to extend the query with words that are semantically related to the given word (*i.e.*, search term). There are various approaches to determine corresponding terms to be added to a query. e.g., semantic networks and thesauruses (see Section 2.5) can be used for this purpose [45,46], as they may describe the *semantic relatedness* of words of a language in graph like structure. Words, which are directly connected to search terms in a corresponding graph, are often good candidates for query expansion.

For a *health recommender system*, but also for recommender systems in general, the findings of IR are indispensable for good recommendation. As shown in Figure 1, *health information artifacts* often come as text and thus, play a similar role as the managed documents of an IR system. The recommendation of such artifacts is based on the user's PHR, which often comprises textual elements such as discharge letters, descriptions of diagnoses, *etc.* These textual elements play a similar role as a query in an IR system. IR techniques to rank documents with respect to a query and to improve term matching can therefore be applied analogously to the task of recommending relevant information artifacts to a PHR user.

#### 2.3.2. Recommender Systems (RS)

In the mid-1990s *recommender systems* have attracted attention from both IT industry and research [15,16,47,48]. *Recommender systems* have become popular especially in the context of online shopping systems in order to recommend purchasable *items of interest* to a shop's users. Typically, the recommendation is given on the basis of a user's former searches or purchases in the shop. Related historical data is stored per user along with his/her account information in order to form a *user profile*. The most popular solution in this context is probably Amazon's suggest service for products stating “Customers Who Bought This Item Also Bought…”.

According to [16] the recommendation problem can be generalized by means of a *utility function u* : *C* × *S* → ℝ, whereby *C* is the set of users and *S* is the set of recommendable items. *u*(*c, s*) returns a real value > 0, where larger values presume a higher interest of *c* in *s* and *u*(*c, s*) = 0 presumes no interest of *c* in *s*. In a sense, *u* is related to the similarity function *sc* from Section 2.3: *c* or respectively *c*'s profile corresponds to a document query *Q* and *s* corresponds to a document *D*.

Initially, *u* is only a partially defined function, where known item ratings are given via users' profiles. e.g., a user *c* might have rated an item *s* explicitly using an online survey or else, the user implicitly expressed interest in the item by purchasing it or by browsing related item information. The goal of a *recommender systems is to extrapolate u* such that *u*(*c, s*) can be computed even for items *s*, which *c* has not yet rated. Ideally *u*(*c, s*) then coincides with the user's true interest in *s*, given he/she actually had to rate it.

According to [16], there are three main approaches to compute *u*(*c, s*) for an item *s*, which user *u* has not yet rated:

1. *Content-based systems compute u*(*c, s*) depending how *c* has rated items similar to *s* in the past. Corresponding high valued items are eventually recommended to the user.
2. Systems using *collaborative filtering* first try to determine other users {*c_i_*}, whose user profiles are similar to that of user *c*. Afterwards, such systems use known ratings for items from users in {*c_i_*} in order to predict *c*'s ratings on these items (given that *c* hasn't rated these items already). Corresponding high valued items are eventually recommended to user *c*.
3. *Hybrid methods* try to combine the former two approaches in some way.

For content-based systems, one needs to assesses the similarity of any two distinct items. If there exists a sufficient textual description for each item, this can be achieved via methods from IR (see Section 2.3.1). However, a user's profile must contain a reasonable amount item ratings already–otherwise no similarity measuring can be performed. This is obviously difficult for new users, whose profile is next to empty; it is known as the *new user problem*.

For systems using collaborative filtering one needs to measure the similarity of user profiles. In this case, it is crucial to have a reasonably large set of well maintained user profiles because otherwise, no users similar to the one under consideration can be found. This problem is known as the *startup problem* and in the case of collaborative filtering, it comes on top of the new user problem.

In the context of an HRS, a user profile is simply an extract or an extension of a PHR. In practice the PHR can be assumed to be filled with quite some entries—otherwise it defeats its purpose. Therefore, *the new user problem is less critical in an HRS*.

We argue that *collaborative filtering is an inappropriate approach for an HRS* because collaborative filtering inspects user profiles *across users*. Considering the high degree of confidentiality required for PHR systems, this would incur too high a security risk. One could argue that not humans but a machine—*i.e.*, the recommendation engine—performs the similarity measuring across user profiles and so, no person will ever get to see, which users are alike. In practice though, it would be very hard to explain this to a standard user, who is worried about the confidentiality of her/his data. Moreover, a technical implementation would have to designed with extreme care in order to avoid security holes: Since data of different users would have to be processed in the same computational units at the same time (*i.e.*, server-side sessions), it might become easier for hackers to exploit potential security holes and to get from one user's profile data to another user's data. Since content-based systems do not hold these problems, *we merely focus on content-based approaches for the rest of this article*. As a fortunate side-effect, this also avoids the above-mentioned startup problem.

### 2.4. Health Related Recommender Systems

Eysenbach was probably the first author who considered ”the linkage of the personal online-accessible health record with general health information from evidence-based resources” [49]. As depicted in Figure 1, this loosely conforms to our suggested combination of a PHR system with an HRS. Several other authors have contributed to this field and therefore their work is discussed briefly in the next paragraphs.

Roitman *et al.* have found that personalized recommendations are a valid approach to increase patient safety by avoiding so-called adverse drug reactions (ADR) [11]. Such ADRs may result from the consumption of several drugs in combination. To avoid such risks the authors suggest to gather information from a patient's PHR and combine it with further information of related web resources (e.g., FDA MedWatch (see: http://www.fda.gov/Safety/MedWatch/)). Thus, recommendations on possible interactions of different drugs are provided for a patient automatically.

A basic form of content recommendation is provided by consumer-centric web portals for medical information, for example symptoms and diseases. Hereby, there is a risk of information overload for laymen. Additionally, it is difficult to offer relevant information “when users have not specified what they exactly want” [12]. However, if users of web portals have an account and a medical profile (*i.e.*, a health record) linked to it, a RS could provide matching *health information artifacts* of higher individual relevance.

Zhang has outlined that the context when investigating for health information plays a major role in understanding and satisfying related information needs of end users [50]. She argued that “people's goals, motivations and emotions” are of vital importance to understand their “cognitive representations of the problem space”. By consideration of these factors, more effective consumer health systems can be designed.

The intention of consumers to use *health recommender systems* seems to be influenced by many factors, for instance by intermediaries which act as an enabler. A study conducted by Wendel *et al.* [51] found that “consumers value [the] usefulness of a [health recommendation] system more and enjoyment less when a general practitioner advices them to use a health recommendation system than if they use it out of their own curiosity”. They emphasize that “communication channels of health recommendation systems […] may be quite difficult to put into practice outside traditional health service channels”. Consequently, there seems to be evidence that such systems should be embedded into existing PHR solutions.

#### 2.4.1. Existing Approaches

Fernandez-Luque, Karlsen and Vognild found “challenges and opportunities” in terms of using recommender technology as a means to educate the uneducated health individual [52]. They suggested the use of so-called Computer-Tailoring Health Education Systems. Moreover, Fernandez-Luque *et al.* described a metric, called *HealthTrust*, to infer information about the trustworthiness of social media content within a health community. They used their method for retrieving videos from the diabetes community on YouTube and found that their approach “performed better than YouTube in nearly all the tested cases” [53].

Morrell and Kerschberg described a system which uses an agent based framework to retrieve content from web resources related to an individual's PHR entries [54]. Semantically related content is presented in a list representation similiar to a search engine and can be used “for further research and consultation”. Thereby, a patient is supported in finding relevant content. The authors emphasize that PHR systems might be considered as a “private enclave” for medical facts. They also suggest the inclusion of an individual's social network to improve the recommendation process.

Rivero-Rodriguez *et al.* outlined a system which enriches media content (*i.e.*, YouTube videos) with content from “very trustful medical sources” like Medline Plus [55]. Their approach leverages the expressiveness of existing ontologies like SNOMED-CT. The system demonstrates that video content can be enriched automatically to increase the level of confidence for patients when dealing with video content from the web. Yet, the authors admit that their method needs improvements in terms of meta-data enrichment to improve the recommendation quality. They also emphasize the importance of a wide range of “content silos of related information”.

Health recommender systems which primarily focus on laymen-friendly textual content are the subject-matter of our research activities. In previous work [56] we proposed a first approach on how to use medical documents and data from a patient's PHR (e.g., discharge letters, medication, etc.) to effectively leverage characteristic information and recommend relevant third party *health information artifacts* to PHR owners. Our implementation of an HRS makes use of a graph data structure related to health concepts derived from *Wikipedia* [57] to compute the individual relevance (see Section 2.5). It only recommends *health information artifacts* originating from knowledge repositories with a high level of trustworthiness (see Section 2.2). Thus, our system supports patients in better understanding their individual state of health in a related medical context. A benefit of using *Wikipedia* as a knowledge resource is that it is available in many localizations for free. We used an HRS-enabled health information system at a public health exhibition which toured throughout Germany and Austria during 2011 [58]. It provided visitors with an insight into relationships of various medical fields in three different languages (German, English, Turkish).

Another approach tries to deliver “lifestyle change recommendations”. Farrell *et al.* outlined an “algorithm for generating retrospective recommendations” [59]. By utilizing only the personal history of an individual, behavioral patterns are extracted and then used as input to the computation of individually tailored recommendations. Yet, the authors admit that the proposed algorithm might suffer from the so-called *new user problem* [60] (see Section 2.3.2). In addition, users of such a lifestyle recommendation system might become dissatisfied with recommended items since these are computed from a user's own temporal activities within the system.

Other systems focus on prevention aspects by delivering recommendations via mobile (smart-) phones. Thereby, patients suffering from chronic diseases (e.g., Diabetes mel.) or people addicted to tobacco receive personalized advice based on their individual (daily) condition [61,62]. Ghorai *et al.* presented a case based recommender system which sends advice to smokers willing to quit. Their approach computes recommendations based on a user's behavioral data which is collected over time.

#### 2.4.2. Evaluation of an HRS

Various methods to evaluate the effectiveness of IR systems exist [63,64]. Often, IR studies use a test collection which serves as a baseline to map queries to items of interest. Yet, most publicly available test collections rely on news paper articles or general news messages and are therefore not especially tailored for the use in the assessment of an HRS (*i.e.*, classic test collections are not derived from electronic medical records which often contain expert terms and medical abbreviations). Hence, in Section 4 we describe the construction of a medical RS test collection (or: gold standard [65,66]) for case-related recommendations.

### 2.5. Semantic Networks

A semantic network is a formal representation of relations among concepts. A related data structure provides means to represent knowledge or to support automated processing for reasoning about knowledge [67].

Nodes represent words and typed edges in the graph express the kind of relatedness of words such as “subordinate term”, “generalizing terms”, “antonym” and so on. There are several electronic resources for semantic networks in different languages such as WordNet [68] or GermaNet [69] that may go way beyond what is needed for query expansion. Alternatively related graphs can be generated from other types of public resources such as *Wikipedia* or large scale RDF-stores such as DBpedia [70].

Semantic networks and ontologies (e.g., SNOMED-CT, OpenGalen [71,72]) have become particularly important in medicine in order to categorize symptoms, diseases, physiological entities, medical procedures and so on. However, these approaches have some clearly noticeable drawbacks. Crafting ontologies can be very time consuming and costly as a group of human experts on a particular domain is needed to do this. Many well known ontologies are often only available in an English version. Such limitation prevents software applications from supporting multi-language enabled semantic computations.

Unfortunately, even less limited approaches such as UMLS [73] do not cover imprecise or colloquial terms. However, these terms are encountered in non-expert vocabulary of PHR system users (e.g., in user initiated queries). This generates impediments to automatic processing, because potentially relevant *health information artifacts* cannot be matched. Moreover, ambiguous abbreviations in clinical text can result in similar problems.

Conceptual structures of publicly available Internet encyclopedias like *Wikipedia* are based on connections among articles, redirects, categories via hyperlinks and hierarchical structures. A generic and automatically generated semantic network covering the health domain to a large extend in different languages was presented in previous work [56]. A so-called Health Graph offers terms only related to medicine and health topics. It is directly derived from a large scale *Wikipedia* snapshot analysis. The resulting Health Graph *G* is directed, weighted and uses the following edge types: *CATEGORY, ARTICLE*, *CODE* (*e.g.*, “E10”) and *REDIRECT, i.e.*, a medical abbreviation or a synonym, e.g., “NSTEMI” to express concept relationships. *G* contains about 350,000 health related nodes and about 4.7 million edges for the English version.

## 3. Concepts

Besides just managing medical and health related data, an HRS-enabled PHR system offers specific information to its users that fits their needs and interests. This can be achieved on the basis of a user's PHR and further profile information, which may have been collected during the user's interaction with the system. As the intention of our approach is to recommend individually tailored health information, an HRS implementation feeds a PHR's UI with a highly specific list of documents relevant to a PHR user's medical history. Hence, one should discuss what data is typically stored about users in a PHR system:

1. explicit medical data about a PHR system user (e.g., current medication, care plans, surgery reports, or discharge letters, *etc.*) such as included in his/her PHR entries
2. terms gathered by the PHR system due to user-initiated searches and queries (e.g., “symptoms myocardial infarction”, “medication flu”)
3. user interaction statistics (*i.e.*, click behavior, duration of page visits, rating of read articles, *etc.*)

In order to enable a viable HRS solution, all of these data may be considered for a recommendation procedure, since they reflect an individual's historical and current health status and potentially his specific health interests. However, so far our suggested approach only considers data according to (1), because it is of predominant importance with respect to a user's information interest.

### 3.1. Challenges & Requirements

When integrating an HRS into a PHR system certain requirements need to be addressed in order to optimize the recommendation:

- The recommendation process must be able to cope with
  (a) imprecise terms (e.g., Hepatitis ⇔ chronic viral Hepatitis),
  (b) colloquial terms (e.g., Period ⇔ Menstruation) and
  (c) misspellings (e.g., Diabedes or Diabedis ⇔ Diabetes mellitus).
- The system must deal with expert vocabulary primarily used by physicians and other health professionals (e.g., Hypo-insulinism ⇔ Diabetes mellitus).
- The system needs to detect whether clinical conditions mentioned in clinical reports are negated. In particular, medical facts (*i.e.*, terms) occur in conjunction with abbreviations frequently used by physicians. In this context, a negation detection algorithm must cope with various negation patterns, for instance:
  (a) “Patient's third ECG and subsequent ECG's show no signs of STEMI” ⇔ excluded term ‘STEMI’
  (b) “Autoimmune retinopathy in the absence of cancer” ⇔ excluded term ‘cancer’
  (c) “Patient suffers from snoring. Analysis did not provide evidence of chronic sleep apnea ⇔ excluded term ‘chronic sleep apnea’
- The confidentiality of PHR user data must be guaranteed under all circumstances. Even administrators of PHR systems must not gain any sort of insight into these data.
- It must also be capable to cope with a new localization of the system, *i.e.*, a change of the user language must be possible without having to manually rework underlying language resources such as text corpora or localized ontologies.

Data entries of medical records are frequently stored as unstructured plain text. This creates further difficulties for IR term matching approaches. A HRS must also recognize expert vocabulary (*i.e.*, common medical abbreviations) and classification system codes primarily used by physicians and other health professionals, such as

- STEMI ⇔ myocardial infarction
- *I21* or *I22* (ICD-10) for ‘myocardial infarction’

Such obstacles can result in less specific recommendations when integrating classic IR approaches into electronic/personal health record systems. Therefore, our approach uses semantic query expansion techniques (see Section 2.3.1) to enrich concept terms found in health record entries to reformulate any query.

Another requirement arises from the classification of *health information artifacts* in terms of laymen-friendliness: Depending on a patient's background knowledge and his/her ability to understand expert documents (or in other words the expert level of a system user) an HRS should be capable of pre-filtering medical information artifacts which might be too difficult to understand for the target reader. This also reduces the number of recommendable candidate documents which have to be processed further and therefore increases computational efficiency.

We address this text categorization problem by training and employing a so-called support vector machine (SVM) in a similar fashion as described in [74]. SVMs originate from the field of machine learning in computer science and are known to perform well even for difficult classification tasks [75]. As basis to use a SVM on text, related documents are first transformed into document vectors according to the vector space model (see Section 2.3.1). On the basis of a larger training set the SVM “learns” to distinguish between vectors of laymen-friendly documents and vectors of expert documents. After the training phase, the classifier can be applied to new documents in order to predict their laymen-friendliness with high accuracy.

### 3.2. Basic HRS Architecture

Our approach tries to recommend personalized *health information artifacts* to be displayed as part of the PHR system's graphical user interface after the user has logged in to the system. In this context, related artifacts can be pre-computed in the background (e.g., once a day by a batch processing job which runs on a regular basis without any user intervention). Thus, an HRS component can deal with increasing amounts of PHR entries.

**Figure 2:**
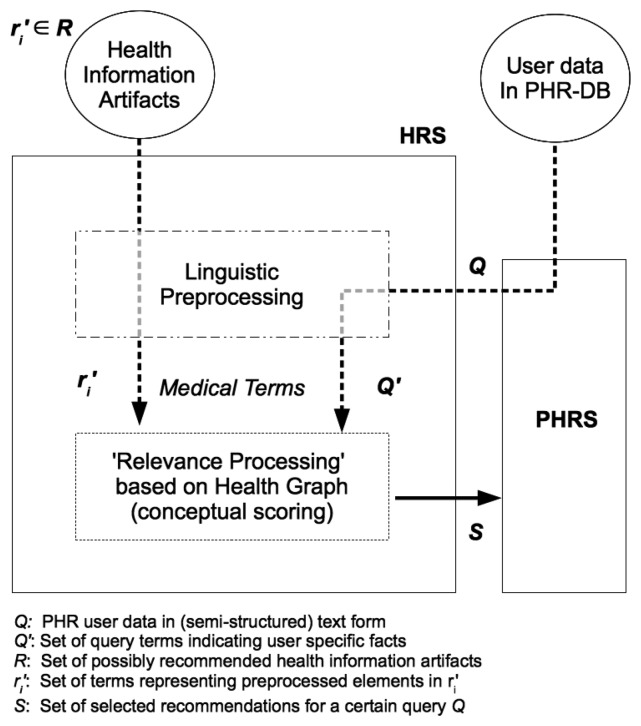
Basic architecture of a proposed HRS. It interacts with a PHR system's database to obtain medical facts to compute individual relevance on the basis of *G*.

By means of linguistic preprocessing, we obtain an extract *Q*′ of the user's PHR data *Q* as input to the relevance processing step (see Figure 2).

The extract *Q′* consist of terms {*q*_1_,…, *q_k_*} originating from the user's PHR data entries but also potentially additional terms as added by semantic query expansion. It is meant to represent the user's medical information interest and serves as a user profile according to Section 2.3.2. Given a set of possible recommendation items *R* = {*r*_1_,…, *r_n_*} (*i.e., health information artifacts*) it is our aim to select those elements in *R* that match best against *Q′.* Thus, a set of relevant recommendations *S* ⊆ *R* is computed.

### 3.3. Refined HRS Architecture

**Figure 3:**
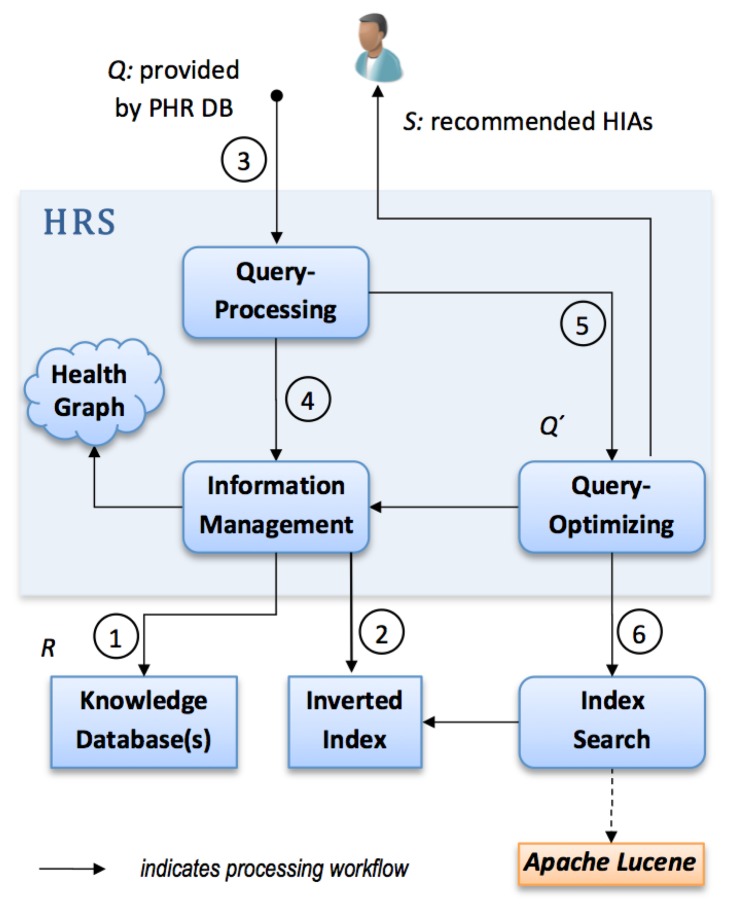
System structure and processing workflow of our HRS prototype and attached data sources. A PHR system feeds in data elements via *Q*. The process yields a set of recommendable items *S* which are highly relevant to a PHR user.

We implemented an HRS prototype as an extension to a PHR system in Java. For this purpose, we introduced a modular structure, as depicted in Figure 3.

At first the HRS prototype connects to one or more knowledge repositories ➀ containing various *health information artifacts* which correspond to *R*. During the next step it creates or updates a so-called inverted index related to all attached knowledge repositories ➁, *i.e.*, it uses indexing techniques from the field of IR (provided by the *Apache Lucene* framework).

Thereafter, our HRS implementation computes a list of relevant *health information artifacts*. *Q* which contains a PHR owner's data entries is used as input to the recommendation process. In ➂ the linguistic query processing module uses a negation detection algorithm that detects whether medical terms in *Q* are present in a negated context. In case negations are found, such terms are removed from *Q* and thus a reduction of terms is achieved. Elements in *Q* might contain ambiguous or misspelled terms not contained in *G*. Therefore, the query processing module performs additional preprocessing on *Q*. In particular, we can rely on Levensthein spelling correction and *n*-gram approximate matching to find equivalent terms which have a semantic context in *G*.

Step ➃ applies semantic query expansion (see Section 2.3.1.) by means of the Health Graph *G*. Access to *G* is provided via the module information management as presented in Figure 3. It selects *k*-nearest neighbours, *i.e.*, nodes connected to a given *q_i_* at a distance of *k*. Thereby, a semantically enriched set of terms *Q′* ≥ *Q* is gained. As context resolution can yield a high number of semantically associated nodes, a module concerned with query optimization reduces the number of terms in *Q′* to a fixed upper limit for simplicity ➄. Relevant concept nodes are selected according to their related edge types. We prefer edge types *REDIRECT* and *CODE* to *CATEGORY* and *ARTICLE*, as they are assumed to have a stronger semantic relationship. The result of the module query optimization is a query string formulated according to *Lucene*'s query parser syntax.

Finally, *Q′* is submitted to the index search module ➅ which retrieves relevant artifacts via the inverted index. The result is a ranked list of recommended documents *S* which is likely to match the health information needs of a PHR system owner.

## 4. Evaluation Approach

The need to evaluate the quality of the proposed approach and a related implementation, mandates a controlled experiment in which a sophisticated system is compared to a naive implementation via a test collection (gold standard). In this section we describe an approach to evaluate such a system via a gold standard in a controlled study involving real world cardiologic cases.

In an initial phase, health professionals will have to assess the relevance of potential matches for a certain clinical case *c_i_*. During this phase a gold standard of human expert recommendations is obtained. The second phase of the evaluation involves at least two implementations of an HRS component:

(1) A *naive HRS implementation* based on well known but basic techniques of IR, in particular on a standard implementation of the VSM using the *Apache Lucene* library. Thus, document sets *D_VSM_* are collected.
(2) An *advanced HRS implementation* which uses query expansion techniques via *G* and features such as negation recognition according to Section 3.3. Thus, document sets *D_HRS_* are collected.

Both implementations will compete against each other in matching the recommendations made by the human experts. Thereby, the retrieval precision *ρ* of both systems will be measured and compared against each other. A statistical test will then reveal which system performed better in computing the ideal set of documents from the content repository *R_c_*. First simulation results indicate that there might be an improvement provided by our advanced HRS implementation, as presented in Section 5.1.

### 4.1. Web-Based Assessment System

**Figure 4:**
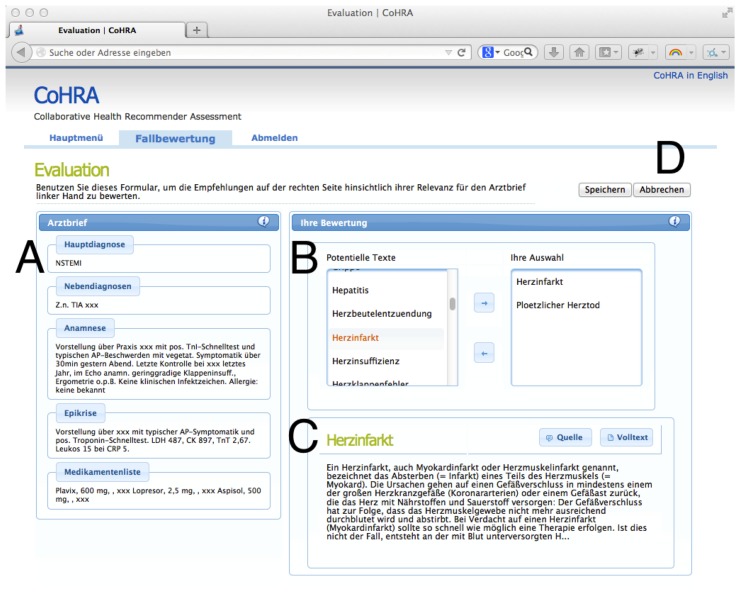
The main view of our HRS assessment system. Health professionals select matching items based on their expertise. To the left (**A**): The cardiologic case (here: NSTEMI/myocardial infarction)—To the right (**B**): Current selections made by an expert (here: ‘Heart attack’ and ‘Sudden cardiac death’).

We implemented a browser-based assessment system, as depicted in Figure 4. In this setting, a cardiologic case *c_i_* ∈ *C* is presented (**A**) on the left.

Every physician selects items from a candidate list (**B**), displayed on the right of the current case. For every candidate item a preview and a full text version is available in the box below the list of items (**C**). Finally, if the health professional has made his/her selection the current case *c_i_* is closed (**D**), selected expert documents are persisted and the next case is presented. Thereby, a set of expert recommendations *D_G_* for all *c_i_* is compiled.

### 4.2. Data Sources

On the one hand, about 27,000 fully anonymized, real-world discharge letters (provided by the *Heidelberg University Hospital*) are available in our letter repository *R_l_*. The letters revolve around the field of cardiology and typically contain text sections including anamnesis, diagnoses, laboratory results, outcomes of procedures and recommended medication. These medical facts represent the foundation for our experiment, as they provide semi-structured text data which has to be processed by any HRS component.

On the other hand, the medical content (*i.e., health information artifacts*) to be rated by the group of physicians is provided by the German Institute for Quality and Efficiency in Health Care *IQWiG* (see: http://www.iqwig.de). It is an independent publisher of evidence-based consumer health and patient information.

This collection of documents comprises a total of about 800 *health information artifacts* written especially for laymen. It is guaranteed to be of high quality and the result of evidence-based medicine. A subset of 75 documents which are relevant for the field of cardiology is presented to the participants of the study.

### 4.3. Setting & Statistical Test

During the next phase of the evaluation (see Section 4), both implementations will compute matching documents pairwise, *i.e.*, for every *c_i_* ∈ *R_l_* we will compute the sets *D_HRS,ci_* and *D_VSM,ci_* . Thereafter, we evaluate the retrieval *precision ρ* of both approaches by computing:

(1a) $$\rho_{H}=\frac{1}{\left|R_{l}\right|}\sum_{c_{i}}\frac{\left|D_{rel,c_{i}}\cap D_{\text{HRS},c_{i}}\right|}{\left|D_{\text{HRS},c_{i}}\right|}$$

(1b) $$\rho_{V}=\frac{1}{\left|R_{l}\right|}\sum_{c_{i}}\frac{\left|D_{\text{rel},c_{i}}\cap D_{\text{VSM},c_{i}}\right|}{\left|D_{\text{VSM},c_{i}}\right|}$$

in which *D_rel_*_,_*_ci_* represents relevant items, *i.e.*, it contains only elements also contained in *D_G,ci_* which originate from recommendations of the test collection previously obtained.

Rather than recommending all relevant documents it is sufficient to deliver only few yet *highly* relevant ones. Thus, as opposed to typical evaluations in IR, we do not consider the fraction of relevant documents that are retrieved (known as *recall* in field of IR).

As we will evaluate a large number of cases (*i.e.*, *n_c_* ≥ 100) a normal distribution can be assumed for this setting. To test if our HRS implementation approach outperforms the basic VSM implementation we postulate: *ρ_H_* > *ρ_V_*. Hence, a dependent *t-Test* for paired samples can be formulated for a one-sided case:

(2) $$\begin{array}{cc} H_{0}:\rho_{H}\leq \rho_{V}, & H_{a}:\rho_{H}>\rho_{V} \end{array}$$

with a significance level of *α* = 0.05 and a target power of 1 − *β* = 0.8. Equation (3) can be reformulated to:

(3) $$\begin{array}{cc} H_{0}:\mu_{H}-\mu_{V}\leq \omega_{0}, & H_{a}:\mu_{H}-\mu_{V}>\omega_{0} \end{array}$$

given that *μ_H_*, *μ_V_* are the mean values of *ρ_H_*, *ρ_V_*. *ω*_0_ represents the expected effect value (*i.e.*, the change in terms of retrieval precision). For the time being we assume an effect size of:

(4) $$\omega_{0}=\Delta \rho =\rho_{H}-\rho_{V}=0.1$$

*i.e.*, an improvement in retrieval precision of 10% is expected.

### 4.4. Sample Size Estimation & Recruitment

A pre-study sample size calculation for *ω*_0_ = 0.1 indicates that a total of *n_c_* = 620 cases (for *ω*_0_ = 0.15 → *n_c_* = 277 and *ω*_0_ = 0.2 → *n_c_* = 156 accordingly) is needed to ensure that a target power of 1 − *β* = 0.8 is achieved.

Yet, there is a certain risk for the data collection being biased (e.g., loss of interest of participants, different levels of expertise, physicians being pressed for time, *etc.*) during phase one of the evaluation. This is especially true if every evaluation case *c_i_* would be assessed by just a single physician. In order to prevent any sort of such biases we plan to assess every *c_i_* at least with an interjudge agreement factor *j* of 2. Thus, the required number of physicians is obtained:

(5) $$n_{p}=\frac{n_{c}*j}{c_{p}}$$

As a consequence, we have to recruit at least *n_p_* = 25 physicians to achieve the target power of 0.8, for a modest *ω*_0_ = 0.1 and the number of cases *c_i_* assessed per participant *c_p_* = 50.

## 5. Results & Discussion

### 5.1. Initial Investigation on Recommendation Quality

Although, the above-described study is not yet completed we found that many phenomena which limit exact term matching (as outlined in Section 3.1.) are indeed present in our collection of discharge letters. We classified health professional language into four categories: *(a) abbreviations; (b) expert terms; (c) colloquial terms; (d) disease codes*.

Table 1 shows a comparison between our advanced HRS implementation and the naive HRS implementation. Each recommended information artifact is ranked according to its normalized score. Note well, that two scores from the two different approaches must not be compared with each other. This originates from the fact, that for our HRS approach *Q* is semantically enriched with a large amount of related terms (see Section 3.3, step ➃).

We used some of the most frequent terms contained in the discharge letters (mentioned in Section 4.2) as input query to compute recommendations. These examples of health professional language are chosen as follows: *(a) NSTEMI*—*non-ST elevation MI; (b) palpitations; (c) Zuckerkrankheit (German term for Diabetes mellitus, frequently used by laymen); (d) I21*—*Acute MI (ICD-10).*

The semantically enhanced HRS approach computes four best matching items within every category. By contrast, the set of recommendations computed by the naive approach contains no elements for category *a* and *d* at all. If elements are found (category *b* and *c*) the relative ranking order varies from the order computed by our HRS implementation:

**Table 1:** Listing of top-4 recommendations: Comparison of recommended information artifacts computed by our advanced HRS implementation and a naive HRS implementation based *Apache Lucene*. Scores normalized to [0..1].

**Category (a) *abbreviations*—primary query term: *NSTEMI***				
**Rank**	**Artifacts (Advanced HRS)**	**Score**	**Artifacts (Naive HRS)**	**Score**
**1**	Coronary disease	1.0	No results at all	-
**2**	Sudden cardiac death	0.915	-	-
**3**	Arteriosclerosis	0.868	-	-
**4**	Myocardial infarction	0.818	-	-
**Category (b) *expert vocabulary*—primary query term: *Palpitations***				

**Rank**	**Artifacts (Advanced HRS)**	**Score**	**Artifacts (Naive HRS)**	**Score**
**1**	Palpitation	1.0	Myocarditis	1.0
**2**	Tachycardia	1.0	Inflammation of heart muscle	1.0
**3**	Cardiac dysrhythmia	0.822	-	-
**4**	Myocarditis	0.802	-	-
**Category (c) *colloquial terms*—primary query term: *Zuckerkrankheit***				

**Rank**	**Artifacts (Advanced HRS)**	**Score**	**Artifacts (Naive HRS)**	**Score**

**1**	Zuckerkrankheit	1.0	Thirst	1.0
**2**	Diabetes mellitus	1.0	Angular cheilitis	0.66
**3**	Prader Willi syndrome	0.573	Prader Willi syndrome	0.583
**4**	Gestational diabetes	0.448	Otitis externa	0.583
**Category (d) *disease codes* (icd-10)—primary query term: *I21***				
**Rank**	**Artifact (Advanced HRS)**	**Score**	**Artifact (Naive HRS)**	**Score**
**1**	Sudden cardiac death	1.0	No results at all	-
**2**	Coronary disease	0.836	-	-
**3**	Arteriosclerosis	0.774	-	-
**4**	Atrial fibrillation	0.762	-	-

A comparison of the result sets (category *b* and *c*) indicates that our HRS offers more relevant results. Apparently, the health information artifact ‘Palpitation’ is not recommended at all by the naive VSM approach, though it is obviously one of the best items that helps to understand ‘palpitations’. This is because no health information artifact contained an entry for the exact term ‘*palpitations*’ in the inverted index. By contrast, our proposed prototype ranks this artifact as its top recommendation along with ‘Tachycardia’, which is a valid medical equivalent. This is made possible by semantic query expansion implemented via the Health Graph *G*.

Category (*d*) illustrates why a regular full-text search is limited. By means of *G* our prototype determines the context of the disease code. Although this disease code never appears in any of the laymen compatible information artifacts, our HRS approach is capable of computing matching artifacts. The naive approach recommends no artifacts at all.

Still, the results presented in Table 1 are only an initial investigation. For this reason, we are conducting the assessment study as outlined in Section 4 at the time of writing.

### 5.2. Limitations of the Study

Unfortunately, there is some uncertainty which results from a yet unknown effect size, *i.e.*, we cannot exactly determine what improvement in retrieval precision can be expected. As this effect size influences the absolute number of cases to be evaluated, this problem makes any prior estimation difficult.

The latter poses a risk to the success of the study, as there might be some dropouts during the recruitment phase already. Consequently, a large effort has to be put into the recruitment phase of physicians which tackle the amount of work to be done. However, there is a chance for an early recalculation of the sample size during phase one if the actual effect size of *ω*_0_ should be higher than initially estimated, e.g., *ω*_0_ = 0.15. Thereby, less physicians would be needed to take part in the evaluation. Moreover, *c_p_* could be lowered or the inter-judge agreement factor *j* could be increased.

Additionally, a bias might occur as the data in *R_l_* originate from the field of cardiology. This might have an effect on how physicians decide which items are relevant. A high number of well selected participants and an accompanying study manual should compensate for this.

### 5.3. Open Issues & Challenges

PHR adoption rates are rising slowly [17]. Yet, many open questions and unresolved issues exist before large real life setting is available. Patient as well as care provider engagement will also play a major role, as a PHRS must be user-friendly for all ages and technological backgrounds. Lack of education in medical terminology and technology skepticism might cause patients to avoid using PHR systems. In particular, concerns about personal health record privacy may be a key factor for the success of (mobile) PHR systems.

PHR systems need well defined international standards to proliferate. Unfortunately, available solutions lack interoperability and inter-system communication is still a future vision. A standardized access interface to PHR entries is crucial for our proposed approach of embedding a *health recommender system* into PHR solutions. A standardized PHR data core including patient and provider identification, basic medications and diagnoses, insurance information, allergies *etc.* would help to avoid the *new user problem* of *recommender systems* as described in Section 2.3.2 Well-suited recommendations, might keep PHR users and care-givers motivated in keeping PHR systems up-to-date. In turn, this may improve recommendation quality because recommendations would be based on highly accurate user profiles.

An open problem for HRSs is to select those entries from the PHR which lead to highly relevant recommendations. In particular, PHR entries referring to a patient's past diseases may be irrelevant or, on the contrary, may well affect the patient's current and future health situation. An HRS should be enabled to assess whether diseases described or encoded in a record entry have long term effects for the patient. e.g., an acute disease like cold that happened a long time ago, can probably be ignored for recommendations in the present. On the other hand, chronic diseases like the diagnosis of diabetes affects a patient at any time and should most likely be considered for recommendations in the present as well as in the future.

## 6. Conclusions

This article has given a general motivation, why there is a need for context-based, individually tailored health information in personal health records. Satisfying this need will help to put patients in control of their own health data and therefore increase patients' autonomy.

An approach of integrating recommender systems into personal health records—termed *health recommender system* (HRS)—was outlined. A first definition of an HRS in the context of personal health record systems was presented. System requirements for such a software component were discussed. We introduced the so-called Health Graph *G*—a graph-based data structure of health related concepts extracted from information included in *Wikipedia*. An HRS prototype which acts as an extension to a PHR system was presented. Given medical facts from PHR data entries, it makes use of techniques like negation detection, spell-correction and semantic query expansion via *G*. Early results indicate an improvement compared to naive approaches from the field of IR.

To evaluate the quality of the proposed approach we designed a controlled experiment in which an advanced HRS implementation is compared against a naive HRS implementation by means of a test collection (gold standard). In a first phase we make use of a group of physicians to develop the test collection. For this purpose, we implemented a web-based assessment system which helps the experts to select laymen-friendly, recommendable documents matching a particular medical case.

A statistical method for sample size calculation was used to estimate how many medical cases should be evaluated to achieve a fixed target power. For a large number of cases, the retrieval precision is computed and a dependent *t-Test* for paired samples will be applied. As a test hypothesis we assume that the retrieval precision of the advanced HRS implementation is significantly higher than the one of the naive HRS implementation. Still, the gold standard does not yet exist—we are currently conducting a recruitment of study participants at different universities and hospitals in Germany and Switzerland.

At the moment, we have only access to a German set of discharge letters and German health professionals; however, a second study based on English PHR documents and a related test collection would desirable to validate the results of the first study.
